# Dorsal Lytic Lesions: Cancer or Infection?

**DOI:** 10.7759/cureus.51993

**Published:** 2024-01-10

**Authors:** Carolina Carreiro, Catarina Gonçalves, Maria Pires, Alexandra Wahnon, Marisa Silva

**Affiliations:** 1 Internal Medicine Department 2, Hospital Santa Maria, Centro Hospitalar Universitário Lisboa Norte, Lisbon, PRT

**Keywords:** vertebral lesions, image-guided biopsy, dorsal pain, spondylodiscitis, bone lytic lesions

## Abstract

Infectious spondylodiscitis is a rare disease and typically presents with an insidious progression characterized by spinal pain that usually starts gradually and progressively worsens over several weeks to months. It occurs through three main mechanisms: direct contamination in cases of trauma or surgery, hematogenous dissemination, or through contiguity.

We report the case of a 63-year-old male, admitted due to a history of dorsolumbar pain after falling from a height of 1.5 meters, with four months of evolution, without other accompanying symptoms, and refractory to anti-inflammatory and analgesic therapy. Initial laboratory evaluation revealed normocytic and normochromic anemia and a slight elevation in C-reactive protein. Computed tomography of the spine showed pathological fractures of T7-T9. A percutaneous biopsy was performed, positive for methicillin-sensitive *Staphylococcus aureus*, and the patient underwent 12 weeks of targeted antibiotic therapy. A surgical procedure with percutaneous posterior arthrodesis from T4 to T12 was performed.

With this case, the authors aim to emphasize the importance of biopsy as a complementary diagnostic method to imaging studies in the diagnosis of spondylodiscitis, with the possibility of identifying the causative agent.

## Introduction

Spondylodiscitis, an infrequent condition, often leads to prolonged hospital stays necessary for its management [1,2]. It involves inflammation within the vertebral bodies and intervertebral discs, typically displaying a slow and subtle progression [1,2]. Despite its rarity, the number of cases has been increasing in the past years [3], highlighting the importance of considering this condition. There is no distinctive symptom, and it presents typically as a non-specific back pain with non-pathognomonic imaging [4], which makes the diagnosis more difficult. While more commonly linked to susceptible populations like older individuals and those with compromised immune systems such as HIV infection or intravenous drug users [4], it is important to be aware that its incidence is rising due to a better understanding of the disease and an improvement in the health care [5]. The diagnosis involves appropriate imaging tests, such as computed tomography and magnetic resonance imaging, urine culture, and serial blood cultures. In the absence of positive cultures and maintaining clinical suspicion, a biopsy should be performed. The authors present a rare case of spondylodiscitis confirmed by biopsy. Currently, the treatment typically leans toward conservative measures involving systemic antibiotics, emphasizing the crucial need for a swift diagnosis to prevent irreversible lesions that may necessitate a surgical approach. 

## Case presentation

**Figure 1:**
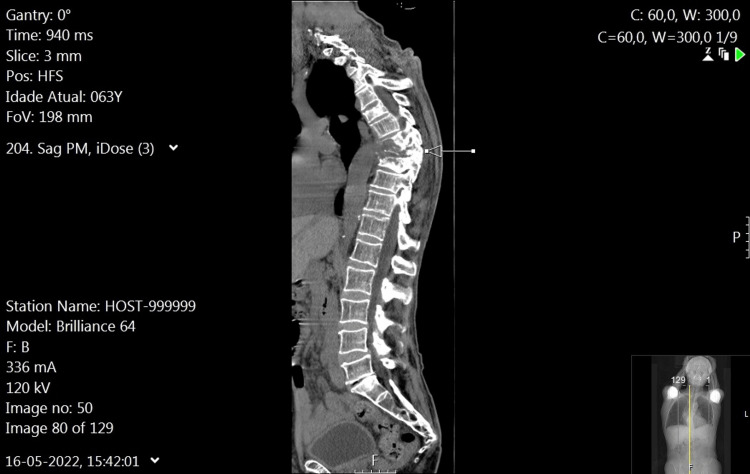
Spinal CT scan (sagittal section) In the sagittal section, a lytic lesion is observed centered at T8, with subtotal destruction of the bodies of T7 and T8, and the upper part of the body of T9. This is associated with erosion of the left pedicle of T8 and the ipsilateral costovertebral joint. There is a marked component with soft-tissue density in the perivertebral region between T6 and T9, with intracanal extension and molding of the adjacent spinal cord surface, and apparent foraminal extension to the right between T6-T7 and T9-T10, and to the left between T7-T8 and T8-T9.

**Figure 2:**
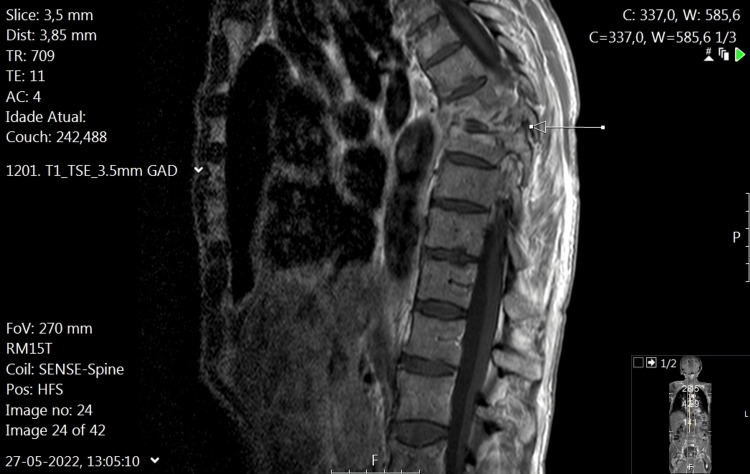
Spinal MRI T2 STIR sagittal section Lytic lesions of T7, T8, and T9 present low signal intensity in T1, high signal intensity in T2 STIR, and heterogeneous post-contrast enhancement; there is marked somatic destruction in D8, with posterior wall retroversion and kyphotic angulation, with slight spinal cord molding. Extensive erosive changes in the vertebral plateaus are associated with high signal intensity in T2 and post-contrast signal enhancement of the D7-D8 and D8-D9 discs; perivertebral lesion component and discreet epidural component are present. Signal changes extend to the posterior arches. STIR, short-tau inversion recovery.

We present the case of a 63-year-old male patient with no relevant personal history, admitted with a four-month complaint of dorsolumbar pain, characterized by mechanical rhythm and radiation to the lower limbs, refractory to analgesic and anti-inflammatory therapy, following a fall from a height of 1.5 meters. He denied any other accompanying symptoms. On physical examination, there were no abnormalities, including sensory-motor deficits or tenderness. Laboratory evaluation upon admission revealed normocytic, normochromic anemia with a hemoglobin level of 10.5 g/dL and a slight elevation in C-reactive protein (2.23 mg/dL; normal range <0.5 mg/dL), without other relevant changes. Computed tomography (Figure 1) and magnetic resonance of the spine (Figure 2) revealed a lytic lesion centered at T8, with subtotal destruction of the bodies of T7 and T8, and the upper part of the body of T9, associated with a soft-tissue density component surrounding T6 to T9, with intracanal extension and molding of the adjacent spinal cord surface, and apparent foraminal extension to the right between T6-T7 and T9-T10, and to the left at T7-T8 and T9-T10. He was admitted for further investigation of lytic lesions in the T spine.

In the context of investigating vertebral lytic lesions, either in the context of metastatic solid tumor or myeloma, a computed tomography scan of the neck, thorax, abdomen, and pelvis was performed, which documented subsegmental right bronchial ectasia with hypodense filling (5 mm), mild bilateral pleural effusion, hepatomegaly with bosselated contours, and small cysts. Bronchoscopy showed no anatomical abnormalities, with bronchoalveolar lavage and bronchial secretions yielding no isolation of microorganisms, including mycobacteria, or the presence of neoplastic cells. Although lytic lesions were not interpreted in the context of prostate cancer, as we were looking for immunosuppression risk factors and because the patient presented a heterogeneous prostate with a periurethral hypercaptating area, most evident in its upper part (12 mm in the axial plane), we performed a transrectal ultrasound that ruled out neoplasia. 

During hospitalization, he developed leukopenia with neutropenia associated with pre-existing anemia, raising suspicion of hematological disease. Myeloma was excluded (protein electrophoresis was normal, as reported in Table 1, beta2 microglobulin was negative). Iron kinetics study revealed a profile of chronic disease anemia without folate or vitamin B12 deficiency, and bone marrow examination showed no infiltration by atypical cells with 3.8% plasmocytes.

**Table 1:** Protein electrophoresis

Protein	Value	Range
Total proteins	6.7 g/dL	
Albumin	3.2 g/dL	3.6-5.4
Alfa1	0.4 g/dL	0.2-0.4
Alfa2	0.9 g/dL	0.45-1.0
Beta1	0.4 g/dL	0.3-0.6
Beta2	0.5 g/dL	0.20-0.53
Gamma	1.4 g/dL	0.71-1.54
Albumin/Gamma globulin ratio	0.9 g/dL	1.15-2.18

He underwent a percutaneous biopsy of the spine, guided by CT, of the T8 lesion via the transpedicular route. The histological examination revealed elements from all three hematopoietic series with maturation, without infiltration by atypical cells. The microbiological examination was positive for methicillin-sensitive *Staphylococcus aureus*. Blood cultures were negative, and transesophageal echocardiography ruled out endocarditis as a source of septic embolization. Although we did not find external injury entrance, as an exclusion diagnosis, Staphylococcal spondylodiscitis was assumed after traumatic fracture of T8, and he completed 12 weeks of antibiotic therapy with flucloxacillin 4 grams/day. He underwent percutaneous posterior arthrodesis from T4-T12 without complications and with resolution of pain. He is still on orthopedics and infectious disease follow-up after 18 months of diagnosis. A year later, a CT scan revealed no abscess or masses. Serial blood cultures after discharge were always negative.

## Discussion

Back pain is a common cause for seeking medical advice. While it is often a routine issue, back pain can occasionally manifest as the sole symptom of vertebral osteomyelitis, an infection that typically targets an intervertebral disc and the two neighboring vertebrae [2]. However, back pain is a symptom that can present in various clinical conditions. Infectious spondylodiscitis is estimated to affect approximately one in 100,000 to one in 250,000 individuals, rendering it still a relatively uncommon clinical condition [1]. This condition exhibits a bimodal distribution, typically impacting patients who are either under 20 years of age or in the age range of 50-70 years [1]. It corresponds to inflammation of the vertebral bodies and intervertebral disc and typically presents an insidious progression. It occurs through three main mechanisms: direct contamination, as in cases of trauma or surgery, hematogenous dissemination, or by contiguity. Risk factors include the presence of infection elsewhere, diabetes, neoplasms, intravenous drug use, and recent surgery (<6 months). The microbial composition depends on the host's risk factors and the specific epidemiological factors in the local area [5]. The most frequently isolated microbiological agents nowadays are *Staphylococcus aureus*, Enterobacteriaceae, commonly *Escherichia coli*, *Mycobacterium tuberculosis*, and *Brucella* spp [1,2]. While hematogenous dissemination remains the primary source of infection, blood cultures often yield negative results [6]. The absence of pathognomonic imaging signals in spondylodiscitis makes its diagnosis challenging [5]. Treatment consists of prolonged antibiotic therapy, with or without surgical intervention, often necessitating extended hospital stays [6,7]. After the discovery of antibiotics, this clinical condition is associated with low morbidity and mortality rates, tends to increase with age, and is largely determined by comorbidities [2].

## Conclusions

With this case, the authors aim to emphasize the importance of biopsy as a complementary diagnostic method to imaging studies in the diagnosis of spondylodiscitis. Although not decisive in the final diagnosis, it can play a significant role in the differential diagnosis. Additionally, it is worth noting that the patient in question had only a history of a fall coinciding with the onset of symptoms, without an evident entry point or risk factor that could explain hematogenous dissemination and *Staphylococcus aureus* fixation in the dorsal spine.
